# Study on prevalence of suicidal ideation and risk factors of suicide among patients visiting psychiatric OPD at Shree Birendra Hospital, Kathmandu Nepal

**DOI:** 10.1371/journal.pone.0254728

**Published:** 2021-07-20

**Authors:** Nagendra Katuwal, Dhan Bahadur Shrestha, Suman Prasad Adhikari, Prakash Raj Oli, Pravash Budhathoki, Richa Amatya, Monalisha Pradhan, Bibhuti Adhikari

**Affiliations:** 1 Department of Neuropsychiatry, Nepalese Army Institute of Health Sciences, Shree Birendra Hospital, Chhauni, Kathmandu, Nepal; 2 Department of Emergency Medicine, Mangalbare Hospital, Morang, Nepal; 3 Department of Internal Medicine, Province hospital, Surkhet, Nepal; 4 Department of Emergency Medicine, Dr. Iwamura Memorial Hospital, Bhaktapur, Nepal; 5 Department of Internal Medicine, Amda Hospital, Damak, Jhapa, Nepal; University of Toronto, CANADA

## Abstract

**Introduction:**

Suicide is a global public health issue. Several environmental, psychosocial, behavioral factors along with physical, sexual, and emotional abuse have been associated with suicidal ideation and attempts. Childhood physical, sexual abuse, and health risk behaviors are also associated with suicidal attempts. The suicidal ideation prevalence varied from 1 to 20% and it varied with study population, geography, age group, gender, and other factors. The Beck suicidal ideation scale is an effective tool for assessing the major suicidal ideation with a six cut-off score.

**Materials and method:**

160 patients who met the inclusion criteria were enrolled into this cross-sectional study after random sampling among the patients visiting the Psychiatric OPD of Shree Birendra Hospital, Kathmandu, Nepal. The Semi-Structured Interview Schedule (SSIS), Beck Scale for Suicide Ideation (BSS), and Kuppuswamy’s Scale were used to collect the data from the patients enrolled in the study. The Chi-square test and binary logistic regression analyses were used to identify and differentiate the factors associated with high suicidal risk.

**Results:**

Out of total 160 patients, 65% (n = 104) were female, 92.5% (n = 148) were married, 61.9% (n = 99) were residing in urban area, 93.1% (n = 148) were Hindus, 74.4% (n = 119) patients were living in the nuclear family, 5% (n = 8) patients had family history of psychiatric illness and 10.6% (n = 17) patients were using the substance of abuse. In the Beck scale for suicidal ideation questionnaire, 87.5% (n = 140) patients had moderate to strong wish to live, 89.4% (n = 143) patients responded as they would take precautions to save a life, 88.8% (n = 142) patients had such ideation/wish for brief, 96.3% (n = 154) had not considered for specificity/planning of contemplated suicidal attempt, 91.9% (n = 147) patients stated that they would not attempt active suicide because of a deterrent example from family, religion, irreversibility of the act and 98.1% (n = 157) patients had revealed ideas of deception/concealment of contemplated suicide openly. 16.9% (n = 27) of participants were categorized as high risk for suicide while 83.1% (n = 133) patients were as a low-risk category for suicide based on the Beck scale for suicidal ideation scoring.

**Conclusion:**

In conclusion, this study found that most of the suicidal attempts were done as an act of impulse and it is higher among female and married individuals residing in the urban areas. This study did not establish any statistically significant association or differences among independent variables with the higher risk scoring in the Beck suicidal ideation scale.

## Introduction

Suicide is a global public health issue. To date, several environmental, psychosocial and behavioral factors are associated with suicidal ideation, suicidal attempts, and suicide in studies conducted in various countries [1]. Physical, sexual, and emotional abuse has been associated with adolescent suicidal ideation and attempts [2]. In addition, childhood physical and sexual abuse appears to be risk factors for future suicide attempts. It is noted that suicidal behaviors often coexist with other health risk behaviors [1, 2]. Recognizing health risk behaviors and other factors that influence suicidal behavior can improve understanding about those at high risk of suicide associated with suicidal behaviors in adolescents. Studies in other countries also revealed that parental support, family factors as well as psychological factors also influence suicidal behavior [3].

It is observed that period prevalence of suicidal ideation varies widely, ranging approximately from 1% to 20% [3]. Also, the prevalence varies with study population, geography, age group, sex, and influence by other factors as mentioned. A study among medical students in Nepal showed suicidal ideation among 10% of students while another study on adolescent students also showed similar findings [4, 5]. In a study conducted among the community and health care seeking populations of five low- and middle-income countries including Nepal showed suicidal ideation in 10.3% [6]. Several risk factors for suicidal behavior have been identified, for example, low socioeconomic status, experienced child abuse, and mental disorders. One of the studies conducted in 2016 at Dharan showed a cut-off score of 6 on Beck’s suicidal ideation scale which is a scale to measure suicidal ideation in this study [7]. Based on a hospital-based study among attempted suicide showed OP poisoning was the most common method [8].

The objective of this study was to study demographic profiles among patients included in the study, meeting the inclusion criteria, and to study the severity of suicidal ideation using the Beck scale among patients having suicidal ideation visiting the psychiatric outpatient department (OPD) of Shree Birendra Hospital (SBH).

## Material and methods

### Study design

The design of the study was a hospital-based cross-sectional study.

### Sampling method

A simple random sampling method was used. Subjects were selected randomly by luck draw among patients visiting the OPD daily. Two number 0 and 1 mentioned in the paper was kept folded in a lucky draw box. Those who received 0 after the lucky draw was not enrolled in the study and those who drew 1 in the lucky draw were enrolled.

### Study site and population

The study site was Psychiatric OPD of Shree Birendra Hospital located in Kathmandu district of Nepal. The study population included patients who visited the Psychiatric OPD of SBH.

### Inclusion criteria

- All patients aged 18–59 years.
- Psychiatric disorders diagnosed (specified in the study objectives) by consultant psychiatrist using ICD-10, DCR
- Literate (with formal schooling/education)
- Those who provided consent to be enrolled in the study

### Exclusion criteria

- Neurodevelopmental Disorders
- Acute Mental Disorders
- Organic mental disorders

### Sample size

The sample size was calculated using a 95% confidence level with ±5% standard error for an unknown population considering a 10% period prevalence for suicidal ideation based on earlier studies in the Nepalese context [6]. The calculated sample size was 139.


$$\begin{array}{l} \text{Calculation},\;\text{n}={\text{z}}^{2}\;(\text{p}.\text{q})\;/\;{\text{e}}^{2}\\ \;=(1.96)2\;(0.1\text{x}0.9)\;/\;(0.05)2 \end{array}$$

The study variables were age, sex, education, personality, socio-demographic profile, psychiatric diagnosis and suicidal Ideation.

### Data collections techniques

Patients who visited the psychiatry OPD were recorded and selected in fixed numbers by lucky draw. Every selected patient was explained about the study and consent was taken in written form. Subsequently, patients were assessed through socio-demographic Pro-forma and self-rated Beck Suicidal Ideation Scale [9]. Data collection was done from July 7, 2020, to Jan 29, 2021.

### Data collections tools

Data collection for the study was done using Semi-Structured interview Schedule (SSIS), Beck Scale for Suicide Ideation (BSS), and Kuppuswamy’s Scale (available as **S1 File**).

### Statistical methods

Data was collected from consented selected patients done using a pre-structured questionnaire, cleaned in Excel, and then imported and analyzed using STATA v.15. Simple descriptive analysis was performed and presented with appropriate frequency tables and cross-tabulation. Kuppuswamy scale, sex, residency of living, marital status and type of the family in which patients were living, family history of psychiatric illness, and psychiatric diagnosis of patients were taken as independent variables while the low or high risk in Beck scale for suicidal ideation taken as the dependent variable.

Logistic regression analysis was performed taking a risk in the Beck scale for suicidal ideation as the dependent variable and other determining socio-economic factors as the independent variable. Sum score of Beck scale for suicidal ideation of > 2 was taken as high-risk category and was the outcome of our interest so logistic regression analysis was run for the high risk for suicidal ideation (> 2) for low risk for suicidal ideation (≤ 2). Firstly, binary logistic regression analysis was used across risk for suicidal ideation for individual independent variables to estimate the unadjusted odds ratio (OR). Then, multiple logistic regression was used to estimate adjusted OR. For logistic regression analysis, high risk for suicidal ideation was labeled as 1 and low risk for suicidal ideation was labeled as 0, and odds of occurrence of high risk for suicidal ideation concerning low risk for suicidal ideation estimated.

### Ethical consideration

The study was carried out after approval from the Institutional Review Committee of the Nepalese Army Institute of Health Sciences (NAIHS). All the participants meeting inclusion criteria consenting for study enrolled in data collection.

## Results

In this study, a total of 160 patients meeting the inclusion criteria were included. There were 65% (n = 104) female and 35% (n = 56) male patients and among them, 92.5% (n = 148) were married. Among 160 patients, 61.9% (n = 99) were residing in urban area and 38.1% (n = 61) patients were in rural area. Out of 160 patients, 93.1% (n = 148) of patients were following the Hindu religion. In the study, the majority of the patients were Chhetri (48.1%) followed by Brahmin (20.6%) and the remaining others were as shown in **Table 1**. 74.4% (n = 119) patients were living in the nuclear family and only 5% (n = 8) patients had a family history of psychiatric illness. About 10.6% (n = 17) patients were using the substance of abuse either alcohol [5% (n = 8)], tobacco/cigarette [5% (n = 8)] or both [0.6% (n = 10)] as shown in **Table 1**. Among 13 different psychiatric diagnoses reported, anxiety disorder not otherwise specified was the commonest diagnosis [36.25% (n = 58)], followed by depression and psychosis [15.63% (n = 25)] each.

**Table 1:** Baseline characters.

Characteristic features		Frequency	Percent
Sex	Female	104	65.0
	Male	56	35.0
Residency	Rural	61	38.1
	Urban	99	61.9
Marital status	Married	148	92.5
	Unmarried	12	7.5
Religion	Buddhism	10	6.3
	Christian	1	.6
	Hinduism	149	93.1
Caste	Brahmin	33	20.6
	Chhetri	77	48.1
	Gurung	4	2.5
	Magar	5	3.1
	Newar	11	6.9
	Tamang	8	5.0
	Others	22	13.8
Family	Joint	41	25.6
	Nuclear	119	74.4
Family history of Psychiatry illness	No	152	95.0
	Yes	8	5.0
Substance abuse	Alcohol	8	5.0
	Alcohol and Tobacco	1	.6
	No	143	89.4
	Tobacco/cigarette	8	5.0
Psychiatric diagnosis	Acute stress reaction	1	0.63
	Adjustment Disorder	7	4.38
	Anxiety Disorder	58	36.25
	Bipolar affective disorder (BPAD)	19	11.88
	Chronic Headache	7	4.38
	Depression	25	15.63
	Epilepsy	2	1.25
	Generalized Anxiety Disorder (GAD)	2	1.25
	Mania	1	0.63
	Obsessive compulsive disorder (OCD)	2	1.25
	Psychosis	25	15.63
	Somatoform disorder	8	5.00
	dissociative Disorder	3	1.88

In the Beck scale for suicidal ideation questionnaire, 87.5% (n = 140) patients had moderate to strong wish to live whereas 10.6% (n = 17) had a weak wish and only 1.9% (n = 3) had no wish to live. In the question regarding the wish to die, 76.3% (n = 122) patients had no wish to die whereas 18.8% (n = 30) had a weak wish to die and only 5% (n = 8) had a moderate to strong wish to die. Among 160 patients, only 4.4% (n = 7) outweigh dying for living while 7.5% (n = 12) equaled the living and dying reason, and the remaining patients gave the reason as living outweigh the dying.

About 3.1% (n = 5) had moderate to strong desire to make active suicide attempts and 8.1% (n = 13) had a weak desire to do so and the remaining had no desire on it. Regarding the passive suicidal desire, 89.4% (n = 143) patients responded as they would take precautions to save a life while 2.5%(n = 4) patients would avoid steps necessary to save or maintain life and remaining would leave life/death to chance. Regarding the duration of having suicide ideation/wish, 88.8% (n = 142) patients had such ideation/wish for brief, fleeting periods. 85.0% (n = 136) patients had suicidal ideation on rarely, occasionally, 88.1% (n = 141) patients were rejecting attitude toward suicidal ideation/wish and 6.3% (n = 10) had ambivalent/indifferent attitude toward it. In addition, 93.8% (n = 150) patients had sense of control over suicidal action/acting-out wish, 5.6%(n = 9) had unsure regarding such control and 0.6% (n = 1) had no sense of control. 91.9% (n = 147) patients stated that they would not attempt active suicide because of a deterrent e.g. from family, religion, irreversibility of the act whereas 5.6%(n = 9) had some concern about deterrents and 2.5% (n = 4) had minimal or no concern about deterrents.

95.0% (n = 152) patients had contemplated the suicidal attempt to manipulate the environment or to get attention/revenge whereas 2.5% (n = 4) had done to escape, surcease, solve problems and remaining patients had contemplated for in-between reasons mentioned as above. 96.3% (n = 154) had not considered for specificity/planning of contemplated suicidal attempt and 93.8% (n = 150) patients had no courage or were a too weak or afraid or incompetent sense of "capability" to carry out the suicidal attempt. Only 1.3% (n = 2) patients had expectancy or anticipation of actual suicidal attempt while 96.3% (n = 154) patients did not expect or anticipate the actual attempt and 2.5% (n = 4) were unsure about it.

Only 0.6% (n = 1) had completed actual preparation for the contemplated attempt whereas 3.1% (n = 5) had partial actual preparation and the remaining patients had not prepared in actual. None of the patients under this study had left suicide notes while contemplating the attempt. 98.8% (n = 158) patients had not made definite plans or completed arrangements for final acts in anticipation of death (e.g., insurance, will) following the suicide attempt while 0.6% (n = 1) had thought about it and made the definite plan. 98.1% (n = 157) patients had revealed ideas of deception/concealment of contemplated suicide openly while 1.9% (n = 3) patients held back such ideas as shown in **Table 2.**

**Table 2:** Beck scale items and rating.

Item and rating		Frequency	Percent	Mean ±SD
1. Wish to live	0. Moderate to strong	140	87.5	0.14±0.402
	1. Weak	17	10.6	
	2. None	3	1.9	
2. Wish to die	0. None	122	76.3	0.29±0.554
	1. Weak	30	18.8	
	2. Moderate to strong	8	5.0	
3. Reasons for living/dying	0. For living outweigh for dying	141	88.1	0.16±0.474
	1. About equal	12	7.5	
	2. For dying outweigh for living	7	4.4	
4. Desire to make active suicide attempt	0. None	142	88.8	0.14±0.432
	1. Weak	13	8.1	
	2. Moderate to strong	5	3.1	
5. Passive suicidal desire	0. Would take precautions to save life	143	89.4	0.13±0.406
	1. Would leave life/death to chance	13	8.1	
	2. Would avoid steps necessary to save or maintain life	4	2.5	
6. Time dimension: Duration of suicide ideation/wish	0. Brief, fleeting periods	142	88.8	0.13±0.390
	1. Longer periods	15	9.4	
	2. Continuous (chronic) or almost continuous	3	1.9	
7. Time dimension: Frequency of suicide	0. Rare, occasional	136	85.0	0.16±0.403
	1. Intermittent	22	13.8	
	2. Persistent or continuous	2	1.3	
8. Attitude toward ideation/wish	0. Rejecting	141	88.1	0.18±0.508
	1. Ambivalent; indifferent	10	6.3	
	2. Accepting	9	5.6	
9. Control over suicidal action/acting-out wish	0. Has sense of control	150	93.8	0.07±0.277
	1. Unsure of control	9	5.6	
	2. Has no sense of control	1	.6	
10. Deterrents to active attempt (e.g., family, religion, irreversibility)	0. Would not attempt because of a deterrent	147	91.9	0.11±0.382
	1. Some concern about deterrents	9	5.6	
	2. Minimal or no concern about deterrents	4	2.5	
11. Reason for contemplated attempt	0. To manipulate the environment; get attention, revenge	152	95.0	0.08±0.347
	1. Combination of 0 and 2	4	2.5	
	2. Escape, surcease, solve problems	4	2.5	
12. Method: Specificity/planning of contemplated attempt	0. Not considered	154	96.3	0.07±0.357
	1. Considered, but details not worked out	1	.6	
	2. Details worked out/well formulated	5	3.1	
13. Method: Availability/opportunity for contemplated attempt	0. Method not available; no opportunity	151	94.4	0.08±0.328
	1. Method would take time/effort; opportunity not readily available	6	3.8	
	2a. Method and opportunity available	3	1.9	
	2b. Future opportunity or availability of method anticipated			
14. Sense of "capability" to carry out attempt	0. No courage, too weak, afraid, incompetent	150	93.8	0.07±0.277
	1. Unsure of courage, competence	9	5.6	
	2. Sure of competence, courage	1	.6	
15. Expectancy/anticipation of actual attempt	0. No	154	96.3	0.05±0.270
	1. Uncertain, not sure	4	2.5	
	2. Yes	2	1.3	
16. Actual preparation for contemplated attempt	0. None	154	96.3	0.04±0.234
	1. Partial (e.g., starting to collect pills)	5	3.1	
	2. Complete (e.g., had pills, loaded gun)	1	.6	
17. Suicide note	0. None	160	100.0	0.00±0.000
	1. Started but not completed; only thought about			
	2. Completed			
18. Final acts in anticipation of death (e.g., insurance, will)	0. None	158	98.8	0.02±0.176
	1. Thought about or made some arrangements	1	.6	
	2. Made definite plans or completed arrangements	1	.6	
19. Deception/concealment of contemplated suicide	0. Revealed ideas openly	157	98.1	0.02±0.136
	1. Held back on revealing	3	1.9	
	2. Attempted to deceive, conceal, lie			

**Fig 1:**
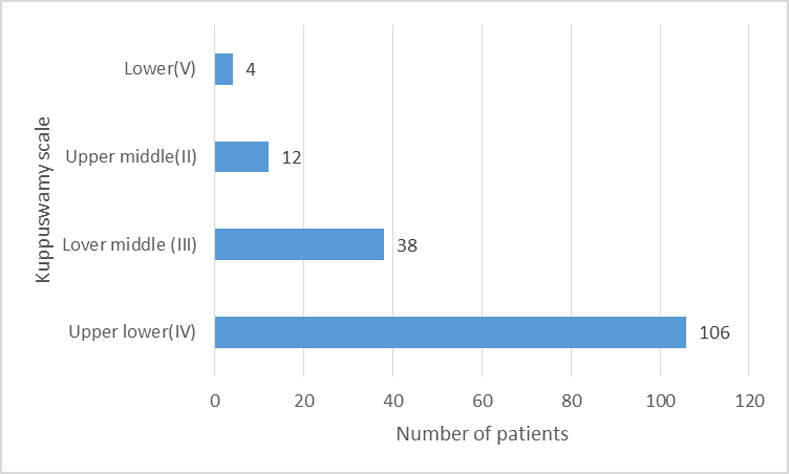
Socio-economic class based on Kuppuswamy scale.

Out of the 160 patients, 66.3% (n = 106) patients belonged to upper-lower class in Kuppuswamy scale, 23.8% (n = 38) belonged to lower-middle class, 7.5% (n = 12) to upper-middle class and 2.5% (n = 4) belonged to lower class in Kuppuswamy scale as shown in **Fig 1**.

**Fig 2:**
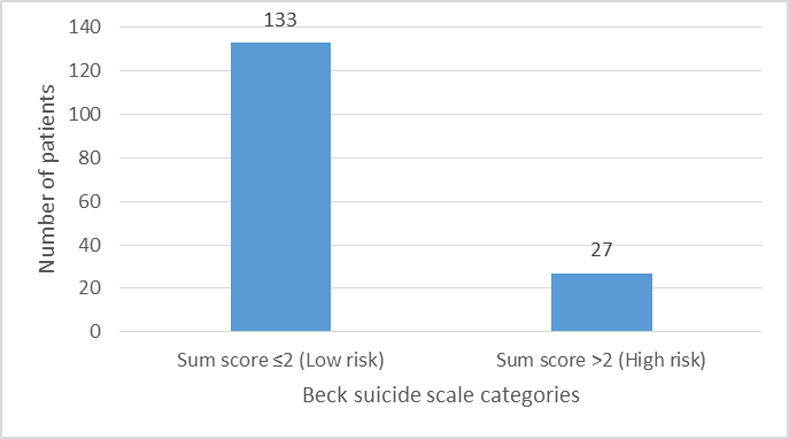
Beck suicidal scale categories for suicide.

In Beck scale for suicidal ideation, out of 160 patients, 83.1% (n = 133) patients had summation of Beck score of ≤ 2 (low risk for suicidal ideation) and 16.9% (n = 27) had summation of Beck score of > 2 (high risk for suicidal ideation) as shown in **Fig 2.**

The Chi-square test was used to check the association among the independent variables and dependent variables (risk for suicidal ideation). From the analysis association between psychiatric diagnosis and Beck scale-based suicide risk category was observed (p<0.05). However, no association was observed between the other independent variables and the dependent variables (Beck scale based suicide risk category) (**Table 3**).

**Table 3:** Cross-tabulation of independent variables across dependent variables using chi-square test.

Variables		Sum score ≤2 (Low risk)	Sum score >2 (High risk)	Total	p-value
Kuppuswamy scale	Lower middle (III)	31(81.58%)	7(18.42%)	38 (100.00%)	0.577*
	Lower (V)	3(75.00%)	1(25.00%)	4 (100.00%)	
	Upper lower (IV)	90(84.91%)	16(15.09%)	106 (100.00%)	
	Upper middle (II)	9(75.00%)	3(25.00%)	12 (100.00%)	
Sex	Female	87(83.65%)	17(16.35%)	104 (100.00%)	0.808
	Male	46(82.14%)	10(17.86%)	56 (100.00%)	
Residency	Rural	53(86.89%)	8(13.11%)	61 (100.00%)	0.319
	Urban	80(80.81%)	19(19.19%)	99 (100.00%)	
Marital status	Unmarried	9(75.00%)	3(25.00%)	12 (100.00%)	0.428*
	Married	124(83.78%)	24(16.22%)	148 (100.00%)	
Family	Nuclear	98(82.35%)	21(17.65%)	119 (100.00%)	0.657
	Joint	35(85.37%)	6(14.63%)	41 (100.00%)	
Mental illness in the family	No	128(84.21%)	24(15.795)	152 (100.00%)	0.134*
	Yes	5(62.50%)	3(37.50%)	8 (100.00%)	
Psychiatric diagnosis	Acute stress reaction	0(0.00%)	1(100.00%)	1 (100.00%)	0.001*
	Adjustment Disorder	3(42.86%)	4(57.14%)	7 (100.00%)	
	Anxiety Disorder	53(91.38%)	5(8.62%)	58 (100.00%)	
	Bipolar affective disorder	16(84.21%)	3(15.79%)	19 (100.00%)	
	Chronic Headache	7(100.00%)	0(0.00%)	7 (100.00%)	
	Depression	18(72.00%)	7(28.00%)	25 (100.00%)	
	Epilepsy	2(100.00%)	0(0.00%)	2 (100.00%)	
	Generalized Anxiety Disorder	1(50.00%)	1(50.00%)	2 (100.00%)	
	Mania	1(100.00%)	0(0.00%)	1 (100.00%)	
	Obsessive compulsive disorder	0(0.00%)	2(100.00%)	2 (100.00%)	
	Psychosis	23(92.00%)	2(8.00%)	25 (100.00%)	
	Somatoform disorder	7(87.50%)	1(12.50%)	8 (100.00%)	
	Dissociative disorder	2(66.67%)	1(33.33%)	3 (100.00%)	

* Fisher’s exact test

Binary logistic regression showed an increase in age lessens the chance of the Beck sum score being >2 (High risk), and diagnosis of adjustment disorder, and depression has higher odds of having Beck sum score being >2 (High risk) in comparison to anxiety disorder not otherwise specified. For the rest of the variables, there were no significant differences in the Beck score category and other independent variables (**Table 4**). Adjusting the effect of one variable over another and rerunning the multinomial logistic regression analysis also showed the persistence of such relation.

**Table 4:** Logistic regression analysis to check relation of Beck score category and other independent variables.

Beck Scale Category	Unadjusted OR	P>|z|	[95% Conf. Interval]		Adjusted OR	P>|z|	[95% Conf. Interval]	
Kuppuswamy scale								
Upper lower®								
Lower(V)	1.476191	0.751	.1329555	16.39	8.480396	0.159	.4319771	166.4836
Upper lower(IV)	0.7873016	0.632	.2962279	2.092456	.9587617	0.952	.2399063	3.831596
Upper middle(II)	1.476191	0.621	.3156051	6.904644	1.144218	0.906	.1211882	10.80332
Sex								
Female®								
Male	1.112532	0.808	.4712804	2.626308	1.524974	0.526	.4144568	5.611066
Residency setting								
Rural®								
Urban	1.573437	0.321	.6422591	3.854683	1.4831	0.494	.4795268	4.586995
Marital status								
Unmarried®								
Married	0.5806448	0.439	0.1463983	2.302952	3.234498	0.273	.396856	26.36214
Family type								
Nuclear®								
Joint	0.8	0.657	.2984636	2.144315	.9868169	0.984	.2636298	3.693845
Age	0.9471994	0.011*	.9082357	.9878348	.9205828	0.010*	.864235	.9806044
Family history of mental illness								
No®								
Yes	3.2	0.128	.7166785	14.28814	2.361642	0.465	.2351647	23.7168
Psychiatric diagnosis								
Anxiety Disorder®								
Acute stress reaction	1				1			
Adjustment Disorder	14.13333	0.003	2.442643	81.77659	18.818	0.004*	2.540391	139.3948
BPAD	1.9875	0.381	.4275044	9.240037	3.495623	0.144	.6534666	18.69932
Chronic Headache	1				1			
Depression	4.122222	0.028	1.162194	14.62124	5.091835	0.025*	1.232128	21.04228
Epilepsy	1				1			
GAD	10.6	0.113	.5719625	196.4465	41.81979	0.030*	1.449263	1206.747
Mania	1				1			
OCD	1				1			
Psychosis	.9217391	0.926	.1664958	5.102851	.9812905	0.983	.1658161	5.807224
Somatoform disorder	1.514286	0.722	.1537855	14.91078	2.647079	0.445	.2177951	32.17256
dissociative Disorder	5.3	0.203	.4057915	69.22274	10.7983	0.103	.6167288	189.0674
_cons					.1595657	0.331	.0039293	6.479756

® = Reference,

*significant taken at p<0.05 with 95% CI, Pseudo R^2^ for multinomial logistic regression model for adjusted OR = 0.1980

## Discussion

This study showed that most of the patients attempting suicide had suicidal ideations for a brief period and on rare occasions with an intact sense of control over the suicidal ideation. In this study, the suicidal attempt was higher among females, 65% of all patients. The study by Sharma B et al. among school-going urban adolescents in Peru also reported higher suicidal attempts among females (53.6%) [1]. The cross-sectional study by Jordans M et al. in five low- and middle-income countries including Nepal had shown that the suicidal attempt was higher among females than males accounting for 45.4% to 79.6% of cases [6]. But the study by Menezes RG et al. done in a medical college in western Nepal showed a male predominance accounting for 54.4% of all cases [5]. In this study, about two-thirds of the patients were from urban areas. Almost (92.5%) all patients enrolled in this study were married and this finding was comparable to the findings from the study by Jordans M et al 2017 in which 69.1% to 91.5% of participants were married [6]. In this study, 93.1% of the participants were Hindus which was higher than the finding from the study done by Menezes RG et al. with 75.7% of Hindu participants [5]. In this study, almost half (48.1%) of the participants were Chettri followed by Brahmin (20.6%) and it could be due to their higher predominance in the general population.

In this study, almost all patients (95%) were without a family history of psychiatric illness. When patients were enquired regarding health risk behaviors, only 10.6% of the patients were abusing the substance. In a study by Sharma B et al in 2015, the health risk behaviors among the participants ranged from 7.0% to 49.2% and were smokers, alcohol drinking, taking illicit drugs, and sexual intercourse [1]. This could be due to the difference among the type of study population.

The majority of the participants in our study wished to live following the act of suicidal attempt with only 5% wishing to die. The majority of the patients in this study were opting for the living with no desire to make active suicide and preferring to take precautions to save a life by avoiding passive suicidal desire. The most common cause for contemplating the suicidal attempt among the study patients was to manipulate the surrounding environment or to get attention or revenge and with secondary gain effect. So, the majority of the patients were having the suicidal ideation/wish for brief, fleeting time and rare or occasionally, lacking the specificity or planning and availability or opportunity of the contemplated attempt, lacking courage, or felt too weak to carry out the attempt. There was a lack of actual preparation for the contemplated act in the majority of the patients and the majority of them did leave the suicide note and did not anticipate the death in the form of insurance, will. Almost all of the patients had concealed their idea of contemplating suicide before their attempt but most of them showed the attitude of rejecting the suicidal ideation/wish. The study by Kim J et al in Korea in 2015 found that the impulsive suicide attempters had shown higher desire to live, less frequency of having suicidal ideation/wish, less pre-occurrence of hopelessness, doing suicidal attempt with a hope to change [10]. This reflects that the majority of the patients enrolled in this study had impulsive suicidal attempts.

Our study showed an increase in age reduces the risk of suicide, however, diagnosis of adjustment disorder, depression, and GAD has higher odds of having the risk of suicide in comparison to anxiety disorder not otherwise specified. Though the statistical relation was observed the confidence intervals were wide due to the small sample of the study, and most of the patients performed suicidal attempts as the act of the impulse. However, our finding of the association of suicidal risk with depression is similar to another study done in eastern Nepal [7]. In the present study, we evaluated some facets of socio-economic, clinical, and behavioral factors for risk of suicide, and the risk of suicide was evaluated using the Beck scale. However, there might be other factors contributing to suicide. So, due to the small sample and limited exploration, further bigger studies exploring more details on the risk of suicide among vulnerable psychiatric patients in the context of Nepal are advised. Additionally, our study is a small size single-center study, so the findings of the present study may vary with others and this needs to be considered while generalizing the findings.

## Conclusion

In conclusion, this study found that most of the suicidal attempts were done as an act of impulse and they were higher among female and married individuals residing in the urban areas. Our study showed an increase in age reduces the risk of suicide. However psychiatric comorbidities like adjustment disorder, depression, and GAD has higher odds of having the risk of suicide in comparison to anxiety disorder not otherwise specified. No statistically significant association or relationship was established among the Kuppuswammy scale, sex, residency, marital status, and type of family with the higher risk scoring in the Beck suicidal ideation scale.

## Supporting information

S1 File Questionnaire. (DOCX)

S2 File Anonymized data set. (DTA)

## References

[1] Sharma B, Nam EW, Kim HY, Kim JK. Factors associated with suicidal ideation and suicide attempt among school-going urban adolescents in Peru. Int J Environ Res Public Health. 2015;12: 14842–14856. doi: 10.3390/ijerph121114842 26610536 PMC4661683

[2] Miller AB, Esposito-Smythers C, Weismoore JT, Renshaw KD. The Relation Between Child Maltreatment and Adolescent Suicidal Behavior: A Systematic Review and Critical Examination of the Literature. Clinical Child and Family Psychology Review. Clin Child Fam Psychol Rev; 2013. pp. 146–172. doi: 10.1007/s10567-013-0131-5 23568617 PMC3724419

[3] Oppenheimer CW, Stone LB, Hankin BL. The influence of family factors on time to suicidal ideation onsets during the adolescent developmental period. J Psychiatr Res. 2018;104: 72–77. doi: 10.1016/j.jpsychires.2018.06.016 29990669 PMC6414226

[4] Casey P, Dunn G, Kelly BD, Lehtinen V, Dalgard OS, Dowrick C, et al. The prevalence of suicidal ideation in the general population: Results from the Outcome of Depression International Network (ODIN) study. Soc Psychiatry Psychiatr Epidemiol. 2008;43: 299–304. doi: 10.1007/s00127-008-0313-5 18264810

[5] Menezes RG, Subba SH, Sathian B, Kharoshah MA, Senthilkumaran S, Pant S, et al. Suicidal ideation among students of a medical college in Western Nepal: A cross-sectional study. Leg Med. 2012;14: 183–187. doi: 10.1016/j.legalmed.2012.02.004 22522041

[6] Jordans M, Rathod S, Fekadu A, Medhin G, Kigozi F, Kohrt B, et al. Suicidal ideation and behaviour among community and health care seeking populations in five low- and middle-income countries: A cross-sectional study. Epidemiol Psychiatr Sci. 2018;27: 393–402. doi: 10.1017/S2045796017000038 28202089 PMC5559346

[7] Pokharel R, Lama S, Adhikari BR. Hopelessness and Suicidal Ideation among Patients with Depression and Neurotic Disorders Attending a Tertiary Care Centre at Eastern Nepal. J Nepal Health Res Counc. 2016;14: 173–179. 28327682

[8] Rawal N, Shrestha DB, Katuwal N, Pathak N. Attempted suicide: Mode and its distribution characteristics among soldiers and their family. J Kathmandu Med Coll. 2018;7: 47–49. doi: 10.3126/jkmc.v7i2.21585

[9] Assessment of suicidal intention: The Scale for Suicide Ideation.—PsycNET. [cited 4 Apr 2021]. Available: https://psycnet.apa.org/record/1979-27627-001 10.1037//0022-006x.47.2.343 469082

[10] Kim J, Lee K, Kim SDJ, … KC-C, 2015 undefined. Characteristic risk factors associated with planned versus impulsive suicide attempters. ncbi.nlm.nih.gov. [cited 4 Apr 2021]. Available: https://www.ncbi.nlm.nih.gov/pmc/articles/pmc4662162/10.9758/cpn.2015.13.3.308 PMC4662162 26598591

