# Supplementary material for: Study on prevalence of suicidal ideation and risk factors of suicide among patients visiting psychiatric OPD at Shree Birendra Hospital, Kathmandu Nepal
Source: PLoS One. 2021 Jul 20;16(7):e0254728. doi: 10.1371/journal.pone.0254728 (PMC8291667; doi:10.1371/journal.pone.0254728)
Supplement: S1 File — (DOCX) [file pone.0254728.s001.docx]

**S1 File**

**Questionnaire**

**Semi structured interview schedule (SSIS)**

***Semi-Structured Pro-forma***

| Age | 18-30, 31-40, 41-50, 51-59 |
| --- | --- |
| Sex | Male, Female, Other |
| Residence | Rural, urban |
| Marital Status | Single, Married |
| Education | Primary Level, Secondary Level, Higher Secondary Level, Bachelor and above |
| Religion | Hindu, Buddhist, Muslim, Christian, Others |
| Caste | Brahmin, Chhetri, Newar, Tamang, Gurung, Kirates Other |
| Occupation | Service, Business, Agriculture, Housewife, Other |
| Type of Family | Nuclear, Joint |
| Number of Family Members | 2, 3, 4, 5, > 5 |
| Monthly Income | <5000, 6000-10000, 11000-15000, 16000-20000, 21000-25000, >25000 |
| Duration of Illness | ………………….years/ months |
| Use of any substance | Yes, No if yes specify…………. |
| Family History of mental illness | Yes, No |

**Kuppuswamy’s Socioeconomic Scale in context to Nepal**

1. **Education score**
2. Professional or Honors………………………………………………….. 4
3. Graduate or Post Graduate……………………………………………… 3
4. High school or Intermediate or Diploma……………………………… 2
5. Illiterate or Primary school ……………………………………… 1
6. **Occupation Score**
7. Legislators, Senior Officials Managers…………………………………. 13
8. Professionals ………………………………………………….. 11
9. Technicians and Associate Professionals …………………………….. 9
10. Clerks ………………………………………………….. 7
11. Service workers and Shop and Market Sales Workers……………….. 6
12. Skilled Agricultural and Fishery Workers ………………….. 5
13. Craft and Related Trades Workers …………………………….. 4
14. Plant and Machine Operators and Assemblers………………………….. 3
15. Unskilled Worker………………………………………………….. 2
16. Unemployed ………………………………………………….. 1
17. **Monthly family income in Rs:**

1) > or = 97451 ………………………………………………….. 12

2) 48751- 97450 ………………………………………………….. 6

4) 24351- 36550 …………………………………………….. 4

3) 114551- 24350 ……………………………………….. 3

2) 4851- 14550 …………………………………………….. 2

1) < or= 4850 …………………………………………….. 1

**Total score Socio-economic class**

26- 29 Upper (I)

16- 25 Upper Middle (II)

11- 15 Middle/Lower Middle (III)

5- 10 Lower/ Upper Lower (IV)

<5 Lower (V)

Beck Scale for Suicide Ideation (BSS)

| Item and rating | |
| --- | --- |
| 1. Wish to live | 0. Moderate to strong |
|  | 1. Weak |
|  | 2. None |
| 2. Wish to die | 0. None |
|  | 1. Weak |
|  | 2. Moderate to strong |
| 3. Reasons for living/dying | 0. For living outweigh for dying |
|  | 1. About equal |
|  | 2. For dying outweigh for living |
| 4. Desire to make active suicide attempt | 0. None |
|  | 1. Weak |
|  | 2. Moderate to strong |
| 5. Passive suicidal desire | 0. Would take precautions to save life |
|  | 1. Would leave life/death to chance |
|  | 2. Would avoid steps necessary to save or maintain life |
| 6. Time dimension: Duration of suicide ideation/wish | 0. Brief, fleeting periods |
|  | 1. Longer periods |
|  | 2. Continuous (chronic) or almost continuous |
| 7. Time dimension: Frequency of suicide | 0. Rare, occasional |
|  | 1. Intermittent |
|  | 2. Persistent or continuous |
| 8. Attitude toward ideation/wish | 0. Rejecting |
|  | 1. Ambivalent; indifferent |
|  | 2. Accepting |
| 9. Control over suicidal action/acting-out wish | 0. Has sense of control |
|  | 1. Unsure of control |
|  | 2. Has no sense of control |
| 10. Deterrents to active attempt (e.g., family, religion, irreversibility) | 0. Would not attempt because of a deterrent |
|  | 1. Some concern about deterrents |
|  | 2. Minimal or no concern about deterrents |
| 11. Reason for contemplated attempt | 0. To manipulate the environment; get attention, revenge |
|  | 1. Combination of 0 and 2 |
|  | 2. Escape, surcease, solve problems |
| 12. Method: Specificity/planning of contemplated attempt | 0. Not considered |
|  | 1. Considered, but details not worked out |
|  | 2. Details worked out/well formulated |
| 13. Method: Availability/opportunity for contemplated attempt | 0. Method not available; no opportunity |
|  | 1. Method would take time/effort; opportunity not readily available |
|  | 2a. Method and opportunity available  2b. Future opportunity or availability of method anticipated |
| 14. Sense of "capability" to carry out attempt | 0. No courage, too weak, afraid, incompetent |
|  | 1. Unsure of courage, competence |
|  | 2. Sure of competence, courage |
| 15. Expectancy/anticipation of actual attempt | 0. No |
|  | 1. Uncertain, not sure |
|  | 2. Yes |
| 16. Actual preparation for contemplated attempt | 0. None |
|  | 1. Partial (e.g., starting to collect pills) |
|  | 2. Complete (e.g., had pills, loaded gun) |
| 17. Suicide note | 0. None |
|  | 1. Started but not completed; only thought about |
|  | 2. Completed |
| 18. Final acts in anticipation of death (e.g., insurance, will) | 0. None |
|  | 1. Thought about or made some arrangements |
|  | 2. Made definite plans or completed arrangements |
| 19. Deception/concealment of contemplated suicide | 0. Revealed ideas openly |
|  | 1. Held back on revealing |
|  | 2. Attempted to deceive, conceal, lie |
